# Using Targeted Liquid Chromatography-Tandem Mass Spectrometry to Rapidly Detect β-Lactam, Aminoglycoside, and Fluoroquinolone Resistance Mechanisms in Blood Cultures Growing *E. coli* or *K. pneumoniae*

**DOI:** 10.3389/fmicb.2022.887420

**Published:** 2022-06-22

**Authors:** Dimard E. Foudraine, Lennard J. M. Dekker, Nikolaos Strepis, Stan J. Nispeling, Merel N. Raaphorst, Wendy Kloezen, Piet Colle, Annelies Verbon, Corné H. W. Klaassen, Theo M. Luider, Wil H. F. Goessens

**Affiliations:** ^1^Department of Medical Microbiology and Infectious Diseases, Erasmus University Medical Center (Erasmus MC), Rotterdam, Netherlands; ^2^Department of Neurology, Neuro-Oncology Laboratory, Clinical and Cancer Proteomics, Erasmus University Medical Center (Erasmus MC), Rotterdam, Netherlands; ^3^Da Vinci Laboratory Solutions, Rotterdam, Netherlands

**Keywords:** liquid chromatography-mass spectrometry, parallel reaction monitoring, antimicrobial resistance, blood cultures, beta-lactamases, GyrA, aminoglycoside resistance, Qnr

## Abstract

New and rapid antimicrobial susceptibility/resistance testing methods are required for bacteria from positive blood cultures. In this study, a multiplex-targeted liquid chromatography-tandem mass spectrometry (LC-MS/MS) assay was developed and validated for the detection of β-lactam, aminoglycoside, and fluoroquinolone resistance mechanisms in blood cultures growing *Escherichia coli* or *Klebsiella pneumoniae* complex. Selected targets were the β-lactamases SHV, TEM, OXA-1-like, CTX-M-1-like, CMY-2-like, chromosomal *E. coli* AmpC (cAmpC), OXA-48-like, NDM, VIM, and KPC; the aminoglycoside-modifying enzymes AAC(3)-Ia, AAC(3)-II, AAC(3)-IV, AAC(3)-VI, AAC(6′)-Ib, ANT(2′′)-I, and APH(3′)-VI; the 16S-RMTases ArmA, RmtB, RmtC, and RmtF; the quinolone resistance mechanisms QnrA, QnrB, AAC(6′)-Ib-cr; the wildtype quinolone resistance determining region of GyrA; and the *E. coli* porins OmpC and OmpF. The developed assay was evaluated using 100 prospectively collected positive blood cultures, and 148 negative blood culture samples spiked with isolates previously collected from blood cultures or isolates carrying less prevalent resistance mechanisms. The time to result was approximately 3 h. LC-MS/MS results were compared with whole-genome sequencing and antimicrobial susceptibility testing results. Overall, there was a high agreement between LC-MS/MS results and whole-genome sequencing results. In addition, the majority of susceptible and non-susceptible phenotypes were correctly predicted based on LC-MS/MS results. Exceptions were the predictions for ciprofloxacin and amoxicillin/clavulanic acid that matched with the phenotype in 85.9 and 63.7% of the isolates, respectively. Targeted LC-MS/MS based on parallel reaction monitoring can be applied for the rapid and accurate detection of various resistance mechanisms in blood cultures growing *E. coli* or *K. pneumoniae* complex.

## Introduction

Rapid identification of bacterial species from positive blood cultures and subsequent rapid antimicrobial susceptibility/resistance testing is vital for early selection of the most appropriate therapy for patients with bloodstream infections (Florio et al., 2018; Lamy et al., 2020). Bacterial species identification can be performed within half an hour after a blood culture becomes positive using one of the available sample preparation methods and subsequent matrix-assisted laser desorption/ionization-time of flight (MALDI-TOF) mass spectrometry analysis (Florio et al., 2018; Kayin et al., 2019). In contrast, antimicrobial susceptibility testing (AST) is still performed using growth inhibition techniques, resulting in a turnaround time (TAT) of 18–48 h, depending on the moment at which the blood cultures become positive, and whether the culture is a pure or mixed culture (Cattoir et al., 2018; Florio et al., 2018). AST TAT can be reduced by collecting bacteria directly from blood cultures, instead of subculturing them first (Kayin et al., 2019). However, even though this results in a significant decrease in TAT, bacteria still require an average of 8–11 h to grow in the presence of antibiotics when using an automated AST system such as the VITEK 2 (bioMérieux) (Machen et al., 2014; Bazzi et al., 2017). AST TAT can be further reduced to approximately 7 h when using the Accelerate Pheno System (Charnot-Katsikas et al., 2018).

In contrast to growth inhibition methods, targeted molecular methods for resistance detection can be applied instantly once blood cultures become positive and provide results within 1–2 h. Commercially available PCR and microarray assays include the BioFire FilmArray BCID2 (BioFire) for the detection of *mecA*, *vanA/vanB*, *bla*_*KPC*_, *bla*_*NDM*_, *bla_*CTX*–*M*_*, *bla*_*VIM*_, *bla*_*IMP*_,*bla_*OXA*–48–*like*_*, and *mcr-1* genes (Cortazzo et al., 2021), and the Verigene BC-GN (Luminex) for the detection of *bla*_*KPC*_, *bla*_*NDM*_, *bla_*CTX*–*M*_*, *bla*_*VIM*_, *bla*_*IMP*_,*bla_*OXA*–23_*, *bla_*OXA*–40_*, *bla_*OXA*–48_*, and *bla_*OXA*–58_* genes (Bork et al., 2015). While these assays already detect around 10 resistance genes, they still miss additional relevant resistance mechanisms, including small-spectrum penicillinases and mechanisms that confer resistance to fluoroquinolones and aminoglycosides.

A future alternative to the use of growth inhibition or gene detection techniques could be the use of targeted liquid chromatography-tandem mass spectrometry (LC-MS/MS). While LC-MS/MS equipment is expensive and not (yet) available in routine diagnostic microbiology laboratories, the technique does offer several advantages compared to other techniques (Charretier and Schrenzel, 2016). Using LC-MS/MS, the actual proteins that confer resistance can be detected instead of their encoding genes. This could especially be useful for the detection of resistance mechanisms of which the quantity is important or resistance mechanisms of which only the detection of genes does not offer enough information. Examples are chromosomal encoded β-lactamases (Rice et al., 2000; Coolen et al., 2019; Han et al., 2020), porins (Nordmann and Poirel, 2019; Vergalli et al., 2020), and efflux pumps (Charretier et al., 2015b; Li et al., 2015; Charretier and Schrenzel, 2016; Cecchini et al., 2018).

Furthermore, targeted LC-MS/MS assays can be highly multiplexed and made suitable for the detection of hundreds of targets in one run (Bourmaud et al., 2016). In addition, by detecting conserved peptides, many protein variants of resistance mechanisms can be detected using single targets (Welker and van Belkum, 2019). In several studies, targeted LC-MS/MS assays were developed for the detection of specific resistance mechanisms such as small-spectrum β-lactamases (Cecchini et al., 2018), carbapenemases (Wang et al., 2017, 2019b; Cecchini et al., 2018; Foudraine et al., 2019; Strich et al., 2019), PBP2a and PBP2c (Charretier et al., 2015a), AmpC β-lactamases (Charretier et al., 2015b), efflux pumps (Charretier et al., 2015b; Cecchini et al., 2018), alterations in the quinolone resistance determining region (QRDR) of GyrA (Hassing et al., 2016), MCR-1 (Wang et al., 2019a), and aminoglycoside resistance mechanisms (Foudraine et al., 2021a). However, the potential of targeted LC-MS/MS to detect resistance mechanisms directly in positive blood cultures has to our knowledge not yet been demonstrated. Furthermore, in the cited studies, assays were developed for a selected number of resistance mechanisms that usually confer resistance to a single class of antibiotics. In this study, we developed and validated a rapid LC-MS/MS assay for the simultaneous detection of 27 prevalent β-lactam, aminoglycoside, and fluoroquinolone resistance mechanisms in blood cultures growing *Escherichia coli* or *Klebsiella pneumoniae* complex.

## Materials and Methods

### Prospective Collection of Blood Cultures

Blood cultures positively tested for Gram-negative rods at the Department of Medical Microbiology in the Erasmus MC were prospectively sampled from 19 October 2019 to 19 July 2020. A volume of 1 ml of each blood culture was pretreated using the Sepsityper kit (Bruker Daltonics, Bremen, Germany) according to instructions of the manufacturer and divided over four aliquots that were stored at −80°C. A total of 144 consecutive cultures were sampled. Thereafter, only repeat samples from the same patients were excluded and cultures containing organisms other than *E. coli* and *K. pneumoniae* complex. A total of 100 samples remained. Of these, 87 samples were pure cultures containing either *E. coli* (*n* = 66), *K. pneumoniae* (*n* = 18), or *Klebsiella variicola* (*n* = 3). The remaining 13 samples were polymicrobial cultures consisting of *E. coli* and Gram-positive cocci (*n* = 4), *E. coli* and other Gram-negative rods (*n* = 2), two different *E. coli* isolates with different AST results (*n* = 1), *E. coli* and *Clostridium perfringens* (*n* = 1), *K. pneumoniae* and other Gram-negative rods (*n* = 3), and *K. variicola* and Gram-positive cocci (*n* = 2). Both pure and polymicrobial cultures were included.

### Spiking of Negative Blood Cultures

To assess the accuracy of LC-MS/MS in detecting less frequently occurring resistance mechanisms, we spiked 148 previously negative blood culture specimens with isolates harboring less prevalent mechanisms of resistance. These were blood cultures that did not yield a positive signal after their standard incubation period and that were therefore regarded as negative. Of these, 100 negative blood cultures were spiked with *E. coli* or *Klebsiella* spp. previously isolated from positive blood cultures that harbored mechanisms conferring resistance to third-generation cephalosporins, aminoglycosides, and/or fluoroquinolones. In addition, 48 blood cultures were spiked with well-characterized isolates carrying resistance mechanisms that are less prevalent in Netherlands. These included 12 *E. coli* isolates and 28 *K. pneumoniae* isolates characterized in a previous study (Foudraine et al., 2021b). Among other resistance mechanisms, these isolates harbored *bla_*CMY*–2–*like*_* (*n* = 8), *bla_*OXA*–48_* (*n* = 7), *bla*_*NDM*_ (*n* = 14), *bla*_*VIM*_ (*n* = 5), *bla*_*KPC*_ (*n* = 5), *aac(3)-IV* (*n* = 2), *aac(3)-VI* (*n* = 3), *ant(2′′)-Ia* (*n* = 5), a*rmA* (*n* = 5), *rmtB* (*n* = 3), *rmtC* (*n* = 4), *rmtF* (*n* = 2), *qnrA* (*n* = 5), and *qnrB* genes (*n* = 9), or they were cAmpC hyperproducers (*n* = 5). Furthermore, eight blood cultures were spiked with seven *Acinetobacter baumannii* isolates (CIP 111171, CIP 111173, CIP 111205, CIP 111208, CIP 111210, CIP 111212, and CIP 111218), and one *E. coli* isolate (CIP 111633) from the Collection de l’ Institut Pasteur (CIP) of the Biological Resource Center of Institut Pasteur (CRBIP, Paris, France) that were also previously characterized and contained various aminoglycoside resistance mechanisms (Ploy et al., 2000; Foudraine et al., 2021a).

Before spiking of negative blood cultures, isolates stored at −80°C were subcultured on Trypticase™ Soy Agar II plates with 5% sheep blood (Becton Dickinson, Franklin Lakes, NJ, United States) and incubated overnight at 37°C. The next day, a 0.5 McFarland suspension was made that was diluted to a concentration of approximately 10^3^ CFU/ml in 0.45% NaCl in water. Subsequently, 1 ml of each suspension was added to a negative blood culture bottle that was incubated overnight using the automated blood culture system BACTEC FX (Becton Dickinson, Franklin Lakes, NJ, United States). The next morning, spiked blood cultures were flagged positive by the Bactec system and were subsequently pretreated in the same way as the prospectively collected blood cultures by making use of the Sepsityper kit.

### Identification and Antimicrobial Susceptibility Testing

Identification and AST were performed at the Erasmus MC for all isolates using subcultures, except for the *Acinetobacter* spp. provided by the CRBIP of which identification data were provided. Identification was performed using the MALDI biotyper (Bruker Daltonics, Bremen, Germany). AST was performed using the VITEK 2 system (BioMérieux, Marcy-l’Étoile, France). An overview of all isolates used for assay validation categorized by their (non-) susceptibility to ampicillin, amoxicillin-clavulanic acid, cefotaxime, ceftazidime, imipenem, meropenem, gentamicin, tobramycin, and ciprofloxacin is shown in Table 1. EUCAST-breakpoints (v 11.0, January 2021) were applied for category interpretation.

**TABLE 1:** Categorization of 248 organisms by antimicrobial susceptibility testing results obtained for different classes of antibiotics.

Collection	Organism	Total (n)	AMP S/NS	AMC S/NS	CTX S/NS	CAZ S/NS	IPM S/NS	MEM S/NS	GEN S/NS	TOB S/NS	CIP S/NS
Prospectively collected organisms	*E. coli*	74	37/37	41/33	65/9	65/9	74/0	74/0	71/3	69/5	56/18
from blood cultures	*K. pneumoniae*	21	0/21	17/4	18/3	19/2	21/0	21/0	21/0	21/0	17/4
	*K. variicola*	5	0/5	5/0	5/0	4/1	5/0	5/0	5/0	5/0	5/0
Previously collected organisms	*E. coli*	73	8/65	13/60	43/30	47/26	72/1	72/1	49/24	41/32	16/57
from blood cultures	*K. pneumoniae*	25	0/25	8/17	9/16	8/17	23/2	23/2	19/6	19/6	5/20
	*K. variicola*	2	0/2	1/1	1/1	1/1	2/0	2/0	1/1	1/1	0/2
Organisms with less prevalent	*E. coli*	13	0/13	0/13	7/6	3/10	11/2	11/2	8/5	7/6	6/7
AMR mechanisms	*K. pneumoniae*	28	0/28	2/26	2/26	2/26	4/24	5/23	2/26	4/24	2/26
	*A. baumannii* *	7	−	−	−	−	−	−	−	−	−

The selected isolates that were previously collected from blood cultures and the selected isolates with less prevalent AMR mechanisms were inoculated in negative blood cultures and subsequently treated in the same way as the prospectively collected blood cultures. Antimicrobial susceptibility testing was performed for all isolates using the VITEK 2. EUCAST breakpoints (v 11.0, January 2021) were applied to categorize isolates as either susceptible (S) or non-susceptible (NS) to ampicillin (AMP), amoxicillin-clavulanic acid (AMC), cefotaxime (CTX), ceftazidime (CAZ), imipenem (IPM), meropenem (MEM), gentamicin (GEN), tobramycin (TOB), and ciprofloxacin (CIP).*Antimicrobial susceptibility testing was not performed for the A. baumannii isolates as the aim was not to predict resistance or susceptibility in A. baumannii.

### Whole-Genome Sequencing and Identification of Antimicrobial Resistance Genes

Whole-genome sequencing (WGS) was performed for the prospectively collected (*n* = 100) and previously collected (*n* = 100) blood cultures by Microsynth AG (Balgach, Switzerland). The remaining 48 isolates were already characterized by WGS. Prior to WGS, extraction, lysis, and DNA isolation were done using the Fast DNA Stool Mini Kit from QIAGEN according to the recommendations of the manufacturer. Bead beating was performed using a FastPrep-24 instrument (MPBiomedicals; 4 cycles of 45 s at speed 4) and 2 ml tubes containing 0.6 g of 0.1 mm glass beads. Next, 200 μl of raw extract was prepared for DNA isolation. For WGS, Illumina DNA Prep libraries (Illumina, San Diego, CA, United States) were prepared according to the instructions of the manufacturer. Subsequently, the libraries were checked for quality and library size on a 2100 Bioanalyzer instrument using a High Sensitivity DNA Assay kit (Agilent Technologies, Santa Clara, CA, United States). The final libraries were quantified using a Quant-iT™ PicoGreen™ ds DNA Assay Kit (Thermo Fisher Scientific, Waltham, MA, United States) and equimolarly pooled prior to sequencing. Sequencing was performed on an Illumina NextSeq 500/550 sequencing system using a NextSeq 500/550 High Output Kit v2.5 for 300 cycles (Illumina, San Diego, CA, United States). Genomes were assembled using Unicycler v0.4 with default parameters (Wick et al., 2017). ABRicate^1^ and the CARD database version 3.0.9 (Alcock et al., 2020) were used to identify the present resistance genes. WGS and analysis of the 48 selected isolates with less prevalent resistance genes was performed previously (Foudraine et al., 2021a,b).

### Assay Development: Selection of Resistance Mechanisms

Most prevalent antimicrobial resistance (AMR) mechanisms were selected that confer resistance to amoxicillin, third-generation cephalosporins, carbapenems, aminoglycosides, and fluoroquinolones in *E. coli* and *K. pneumoniae*. The β-lactamases SHV, TEM, OXA-1-like, CTX-M-1-like, CMY-2-like, chromosomal *E. coli* AmpC (cAmpC), OXA-48-like, NDM, VIM, and KPC were selected. Furthermore, the AMEs AAC(3)-Ia, AAC(3)-II, AAC(3)-IV, AAC(3)-VI, AAC(6′)-Ib, ANT(2′′)-I, and APH(3′)-VI were selected, and the 16S-RMTases ArmA, RmtB, RmtC, and RmtF. For fluoroquinolone resistance, the mechanisms AAC(6′)-Ib-cr, QnrA, QnrB, and the wildtype QRDR of GyrA were selected. Finally, for *E. coli*, the porins OmpC and OmpF were selected. In total, 27 different antibiotic resistance mechanisms were selected.

### Assay Development: Peptide Selection

Specific peptides were selected for the detection of each resistance mechanism and were included in the assay. Peptides for the carbapenemases (Foudraine et al., 2019), AMEs and 16S-RMTases (Foudraine et al., 2021a), GyrA (Hassing et al., 2016), and for CMY-2-like, *E. coli* cAmpC, and the *E. coli* porins OmpC and OmpF (Foudraine et al., 2022)were selected from previous studies. In addition, three previously selected internal quality control peptides were included (Foudraine et al., 2021a). For SHV, TEM, OXA-1-like, CTX-M-1-like, QnrA, and QnrB, candidate peptides were first selected *in silico* using the same method that was previously described in detail (Foudraine et al., 2019, 2021a). Thereafter, all candidate peptides were tested experimentally and the best-performing peptides with the highest intensity and low interference were included in the assay. The aim was to include at least two peptides for each resistance mechanism. Candidate peptides were tested in two to four isolates for each resistance mechanism using a set of previously characterized isolates (Foudraine et al., 2021b). *In silico* specificity analysis was performed for all newly selected peptides using the NCBI non-redundant protein sequence database and protein BLAST. The final selection of peptides used for assay validation is shown in Table 2. Stable isotope labeled (SIL) peptide variants of all peptides were included for assay optimization as previously described (Foudraine et al., 2019, 2021a). SIL peptides were synthesized by Pepscan (Lelystad, Netherlands).

**TABLE 2:** Selected target peptides included in the final panel for the detection of 27 resistance mechanisms and three internal quality control proteins.

Protein	Peptide	Misses variants	Charge	Mass (m/z)	Retention window (min)	Fragment ions included	Concentration SIL peptides in digest (fmol/μ l)
SHV	GIVALLGPNNK	SHV-75, −103, −137, −150, and 9 anonymous	2+	548,32967	5.43–7.43	y8, y5, b3	5,0
SHV	VDAGDEQLER	SHV-15, −135, −156, and 4 anonymous	2+	566,26747	2.16–4.16	y9, y7, y6, y4	5,0
TEM	SALPAGWFIADK	TEM-60, −147, −167, −176, −183, and 4 anonymous	2+	638,34024	7.17–9.17	y9, y8, y7, y5, y4	2,5
TEM	QIAEIGASLIK	TEM-101, −104, −151, −164, and 6 anonymous types	2+	571,84261	5.74–7.74	y9, y7, y6, y4	2,5
CTX-M-1-like	QLGDETFR	CTX-M-68, −177, and 3 anonymous	2+	483,23798	3.17–5.17	y7, y6, y5, y4, y3	10,0
CTX-M-1-like	AQLVTWMK	4 Anonymous	2+	488,76786	5.34–7.34	y6, y5, y4, b3	10,0
OXA-1	TLQNGWFEGFIISK	None	2+	820,42757	8.88–10.88	y11, y10, y6, y3	10,0
OXA-1	TGAGFTANR	1 Anonymous	2+	447,72504	1.65–3.65	y8, y7, y6, y5, b3	10,0
CMY-2-like	TFNGVLGGDAIAR	#	2+	645,84368	5.23–7.23	y11, y10, y9, y8, y7	5,0
CMY-2-like	TGSTGGFGSYVAFVPEK	#	2+	852,41740	6.68–8.68	y13, y12, y10, y6, y4	5,0
Ec − cAmpC	SSSDLLR	#	2+	389,20869	2.4–4.4	y6, y4, y3	5,0
Ec − cAmpC	QPVTQQTLFELGSVSK	#	2+	881,47271	6.68–8.68	y11, y10, y8, y6, y5	5,0
KPC	GFLAAAVLAR	None	2+	494,80055	6.48–8.48	y8, y7, y6, y5, y4	10,0
KPC	APIVLAVYTR	None	2+	551,83459	5.66–7.66	y8, y7, y6, y5, b3	10,0
OXA-48-like	SQGVVVLWNENK	OXA-199 en 3 anonymous	2+	686,86461	5.54–7.54	y8, y7, y6, y5, b3	5,0
OXA-48-like	ANQAFLPASTFK	+	2+	647,84314	5.31–7.31	y9, y8, y7, y6, b3	5,0
NDM	AFGAAFPK	None	2+	404,72124	4.09–6.09	y6, y5, y4, y3, b3	2,5
NDM	FGDLVFR	+	2+	427,23197	5.88–7.88	y6, y5, y4, y3, b3	2,5
VIM	NTAALLAEIEK	+	2+	586,82970	6.28–8.28	y9, y8, y7, y6, y5	2,5
VIM	VGGVDVLR	+	2+	407,74270	3.52–5.52	y7, y6, y4, y3, b3	2,5
ArmA	VATLNDFYTYVFGNIK	*	2+	932,97763	9.77–11.77	y12, y9, y8, y5, b3	5,0
ArmA	VIGNELVYITSGFQK	*	2+	834,45379	7.99–9.99	y13, y10, y9, y8, y6	5,0
RmtB	ILTEEWGR	*	2+	502,26400	4.36–6.36	y7, y6, y5, y4, y3	5,0
RmtB	ALSLHASTK	*	2+	464,26654	1.79–3.79	y7, y6, y5, y4, y3, b3	5,0
RmtC	ILNLHTSTNER	*	2+	649,34658	2.62–4.62	y10, y9, y6, y5, b3	2,5
RmtC	NAVISFPIK	*	2+	494,79494	5.98–7.98	y7, y6, y5, y3, b3	2,5
RmtF	LLPVLEAQK	*	2+	505,81587	5.02–7.02	y6, y5, y4, y3	2,5
RmtF	LVVTFPTR	*	2+	466,78183	5.08–7.08	y7, y6, y5, y3	2,5
AAC(3)-Ia	AALDLFGR	*	2+	431,74270	6.13–8.13	y7, y6, y5, y4, y3	2,5
AAC(3)-Ia	LGPDQVK	*	2+	378,71615	1.81–3.81	y6, y5, y4, y3	2,5
AAC(3)-II	AIGPVEGGAETVVAALR	*	2+	805,44904	7.3–9.3	y13, y12, y11, y10, y9	5,0
AAC(3)-II	GFGLLNQFLVQAPGAR	*	2+	844,46756	9.09–11.09	y11, y9, y8, y7, y4	5,0
AAC(3)-IV	VPYGVPR	*	2+	394,22669	2.67–4.67	y6, y5, y4	2,5
AAC(3)-IV	EGPVGHAFAR	*	2+	520,76724	2.12–4.12	y7, y6, y5, y4, y3	2,5
AAC(3)-VI	KNGDLHEPATAPATPWSK	*	3+	640,65852	3.41–5.41	y10, y9, y8, y5, y4	2,5
AAC(3)-VI	VAQGPVGGAQSR	*	2+	563,80181	1.27–3.27	y9, y8, y7, y6, y5	2,5
AAC(6′)-Ib	ALVELLFNDPEVTK	*	2+	794,43506	8.37–10.37	y9, y8, y7, y5, b4	5,0
AAC(6′)-Ib	IQTDPSPSNLR	*	2+	614,32003	2.67–4.67	y9, y8, y7, y6, y5	5,0
AAC(6′)-Ib-cr	QGTVTTPYGPAVYMVQTR	*	2+	984,99603	6.08–8.08	y13, y10, y9, y6, b3	5,0
ANT(2′′)-I	NLPLWIGGGWAIDAR	*	2+	819,94118	9.4–11.4	y13, y11, y10, y9, b3	12,5
APH(3′)-VI	AGLADEFVDISFVER	*	2+	834,41740	8.87–10.87	y11, y9, y8, y7, y6	2,5
APH(3′)-VI	IGQSPSDVYSFNR	*	2+	735,35461	4.38–6.38	y10, y9, y8, y6, y5	2,5
GyrA	YHPHGDSAVYDTIVR	Not applicable	2+	865,41827	3.48–5.48	y12, y11, y9, y8	12,5
QnrA	VNLEGVK	None	2+	379,72398	2.49–4.49	y6, y5, y3	2,5
QnrA	VFQQEDFSR	None	2+	578,27510	3.32–5.32	y7, y5, y4, y3	2,5
QnrB	GASFMNMITTR	QnrB72, 74 and 96	2+	614,79428	6.55–8.55	y8, y7, y6, b3	2,5
QnrB	GVDLQGVK	QnrB2, 13, 20, 22, 31, 47, 49, 64	2+	408,23471	2.76–4.76	y6, y4, b3	2,5
Internal control protein–Chaperon protein DNAK	SLGQFNLDGINPAPR	Not applicable	2+	799,91790	6.66–8.66	y11, y10, y8, y7, y4	12,5
Internal control protein–30S ribosomal protein	GATVELADGVEGYLR	Not applicable	2+	775,39647	6.88–8.88	y11, y10, y9, y8, y7	12,5
Internal control protein–DNA direct RNA polymerase	VADLFEAR	Not applicable	2+	460,74544	4.53–6.53	y7, y6, y4, y3, b3	5,0
OmpC	VAFAGLK	#	2+	353,21833	3.77–5.77	y6, y5, y4, y3	5,0
OmpC	VGSLGWANK	#	2+	466,25343	3.51–5.51	y8, y7, y5, y4, y3	5,0
OmpC	FQDVGSFDYGR	#	2+	645,79111	4.81–6.81	y9, y8, y7, y3, b3	5,0
OmpC	GNGFATYR	#	2+	443,21431	2.54–4.54	y6, y5, y4, y3, b3	5,0
OmpF	AVGLHYFSK	#	2+	511,27691	3.54–5.54	y7, y5, y4	5,0
OmpF	VGGVATYR	#	2+	411,72705	1.94–3.94	y7, y6, y4, y3, b3	5,0

+As previously reported (Foudraine et al., 2019).# As reported (Foudraine et al., 2022).* As previously reported (Foudraine et al., 2021a).“Anonymous” variants were unique β-lactamase variants similar to the reference sequence which were not (yet) annotated at the time of peptide selection.

### Assay Development: Concentration of Stable Isotope Labeled Peptides and Detection Criteria

Based on pilot experiments, concentrations of SIL peptides were experimentally set to approach the concentrations of the endogenous peptides (Table 2). In addition, a set of detection criteria was established before assay validation to automate and standardize peptide detection. The following criteria were set, namely, mass error <10 ppm, rdotp >0.95, ratio of endogenous to SIL peptide >0.01. For SHV, the ratio to SIL peptide >0.01 criterion was not applied as the range of ratios for positive samples in the pilot experiments included ratios lower than 0.01. For the selected AAC(6′)-Ib(-cr) peptides, the ratio to SIL peptide was increased to a cutoff ratio of >0.05 to prevent false-positive detection due to carryover. Proteins were considered detected when at least one peptide of the protein met the selected criteria.

### Assay Development: (Non-)susceptibility Prediction Criteria

To predict whether the isolates were susceptible or non-susceptible based on the detected peptides, the following prediction rules were applied. Both *K. pneumoniae* and *K. variicola* were regarded as intrinsically resistant to ampicillin.^2^ Presence of TEM or SHV was predicted to only result in non-susceptibility to ampicillin (Bush and Jacoby, 2010). Presence of OXA-1 was predicted to result in non-susceptibility to ampicillin and amoxicillin-clavulanic acid (Ortega et al., 2012; Evans and Amyes, 2014). Presence of CTX-M-1-like was predicted to result in non-susceptibility to ampicillin and to third-generation cephalosporins (Bush and Jacoby, 2010). Presence of CMY-2-like or cAmpC was predicted to result in non-susceptibility to ampicillin, amoxicillin-clavulanic acid, and third-generation cephalosporins (Bush and Jacoby, 2010). Presence of OXA-48-like was predicted to result in non-susceptibility to ampicillin, amoxicillin-clavulanic acid, meropenem, and imipenem (Walther-Rasmussen and Hoiby, 2006). NDM, KPC, and VIM were expected to result in non-susceptibility to all of the tested β-lactams (Walther-Rasmussen and Hoiby, 2006). For aminoglycosides, the same prediction rules were applied as reported previously (Foudraine et al., 2021a). Presence of QnrA, QnrB, or AAC(6′)-Ib-cr, or absence of the wildtype QRDR of GyrA were all expected to result in non-susceptibility to ciprofloxacin (Jacoby, 2005; van der Putten et al., 2019).

### Lysis and Digestion Protocol

For assay validation, stored Sepsityper pellets (i.e., positive blood cultures were pretreated by making use of the Sepsityper kit) were suspended in 50 μl lysis solution consisting of 5% sodium deoxycholate, 7.5 mM dithiothreitol in water. Thereafter, SIL variants of all included peptides were added. Next, samples were sonicated for 5 min using a Branson 2510 Ultrasonic Cleaner (Branson Ultrasonics, Danbury, CT, United States) and incubated for 10 min at 80°C and 450 rpm in a heater shaker (Eppendorf ThermoMixer C, Eppendorf, Hamburg, Germany). Subsequently, samples were diluted by adding 450 μl MS grade water and 7.5 μl trypsin (1 μg/μl) (Worthington, NJ, United States) was added followed by 20 μl Tris-HCl buffer (50 mM, pH 8). Next, samples were incubated for 1 h at 37°C and 450 rpm. To stop the protein digestion, 20 μl of 5% trifluoroacetic acid was added and samples were incubated for 5 min at 37°C and 450 rpm. Finally, digests were centrifuged for 10 min at 4°C and 21,000 × *g*, and 250 μl of supernatant was transferred to Eppendorf tubes and stored at -20°C.

### Liquid Chromatography-Tandem Mass Spectrometry

For each sample, 20 μl of digest was loaded onto Evotips (Evosep, Odense, Denmark) according to the instructions of the manufacturer and as previously described (Bache et al., 2018; Foudraine et al., 2019). LC-MS/MS was performed using the Evosep One (Evosep, Odense, Denmark) coupled to an Orbitrap mass spectrometer (Q Exactive HF Hybrid Quadrupole-Orbitrap, Thermo Fisher Scientific, Bremen, Germany). LC was performed using the separation method of the manufacturer of 11.5 min (100 samples/day) (Bache et al., 2018). The Q Exactive HF system was operated in PRM mode. Samples were sequentially measured and the column was not rinsed with a blank in between measurements. The following settings were used during method validation, namely, a quadrupole isolation window of 0.6 *m/z* units, an automatic gain control target value of 1 × 10^6^ ions, a maximum fill time of 150 ms, and a resolving power of 15,000 at 400 *m/z*. A normalized collision energy of 27% was used for all peptides. A retention time window of 2 min was used for each peptide.

### Data Analysis

Skyline daily version 20.1 or later was used for MS analysis (MacCoss Lab Software, University of Washington, Seattle, WA, United States). After assay development, Skyline results were analyzed using a custom-made software tool “Antibiotic Resistance Scanner” developed by Da Vinci Laboratory Solutions (Rotterdam, Netherlands). By using this tool, Skyline results were imported and evaluated in an automated fashion by the applied detection criteria. Test characteristics, i.e., the sensitivity, specificity, and the corresponding 95% CIs were calculated using VassarStats: Website for Statistical Computation.^3^

## Results

**FIGURE 1:**
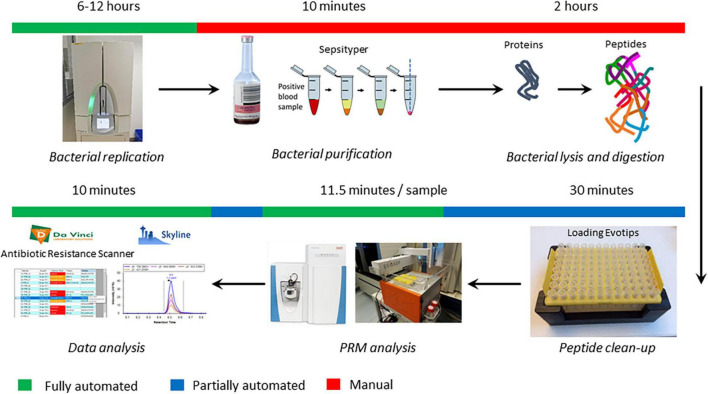
Overview of the current protocol from positive blood culture to results. For each step, the approximate required time is indicated as well as the level of automation. PRM, parallel reaction monitoring.

**FIGURE 2:**
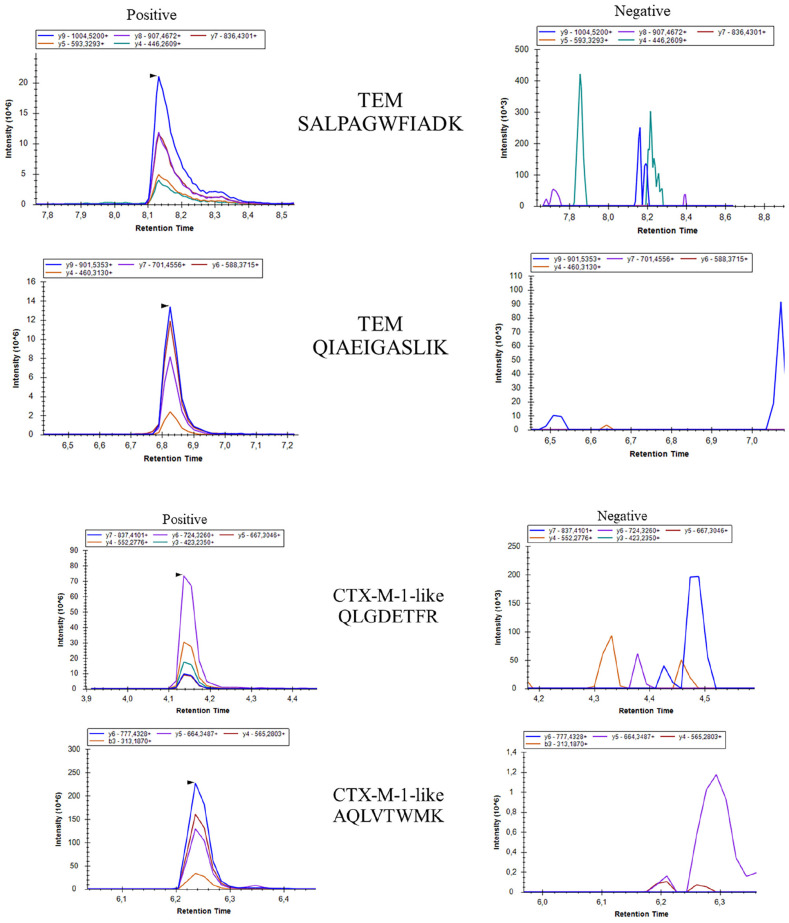
Examples of positive and negative LC-MS/MS spectra of peptides selected for TEM and CTX-M. On the *x*-axis, the retention time in minutes, on the *y*-axis the signal intensity in unadjusted units rescaled to show clear spectra, or a lack thereof in the negative samples.

In previous studies, we demonstrated the possibility to detect different carbapenemase enzymes and aminoglycoside resistance mechanisms with high accuracy using LC-MS/MS (Foudraine et al., 2019, 2021a). In these studies, resistance mechanisms were identified by detection of selected specific peptides, and SIL peptides were used to automate the analysis. In addition, internal quality control peptides were used to assess sample quality and automate analysis. In this study, similar conditions were applied to detect these and additional resistance mechanisms in positive blood cultures growing either *E. coli* or *K. pneumoniae* complex isolates, thereby omitting a re-culture/isolation step prior to resistance testing that results in a significant decrease in time to result (Figure 1). To expand the coverage of resistance mechanisms, TEM, SHV, CTX-M, OXA-1 and QnrA and QnrB peptides were added to previously selected targets (Table 2). Examples of LC-MS/MS spectra of TEM and CTX-M are shown in Figure 2. In pilot experiments, we verified that all 27 selected resistance mechanisms could be detected within a gradient of 11.5 min. Under these conditions, all 248 isolates were tested.

### Assay Validation: Quality Assessment and Sample Exclusion

Quality control parameters were set to exclude samples that were deemed of insufficient quality. Minimum requirements were that one SIL peptide was detected for each protein, and that all three internal quality control peptides were detected (for *E. coli* and *Klebsiella* sp.). Of the 248 samples, three prospectively collected samples (of which two mixed cultures) and one spiked sample (that also required a more extensive extraction protocol prior to identification using MALDI-TOF) were excluded as multiple SIL peptides were missed by both initial and repeated measurements. In addition, three other prospectively collected samples (of which one mixed culture) were excluded in which at least one of the internal quality control peptides was not detected. Therefore, 241 samples were used for analysis.

### Assay Validation: Comparison of Mass Spectrometry Results to Whole-Genome Sequencing Results

Detection of resistance mechanisms by LC-MS/MS was compared to the results obtained by WGS for all 241 remaining samples. The results are shown in Table 3. For the β-lactamases CTX-M-1-like, OXA-1, CMY-2-like, and for the four carbapenemases KPC, OXA-48, NDM, and VIM, the sensitivity was 100% compared to WGS analysis. The β-lactamase TEM was detected in 104 of the 106 isolates harboring TEM genes. SHV β-lactamases were not detected in 7 of the 66 isolates carrying SHV genes. The chromosomal-encoded AmpC β-lactamase was detected in 11 of the 155 *E. coli* isolates, which is to be expected as chromosomal encoded *bla*_*ampC*_ is often not or minimally expressed (Livermore, 1995; Jacoby, 2009). In addition to a high sensitivity for the included β-lactamases, the assay was 100% specific for CTX-M-1-like, OXA-1, NDM, and VIM, and 96.6% for SHV, 95.6% for TEM, 98.7% for CMY-2-like, 99.1% for KPC, and 99.6% for OXA-48.

**TABLE 3:** Sensitivity and specificity of LC-MS/MS compared to WGS in detecting the selected resistance mechanisms in 241 isolates.

Resistance mechanism	Number of MS positive/number of WGS positive isolates	Sensitivity (95% CI)	Number of MS negative/number of WGS negative isolates	Specificity (95% CI)
SHV	59/66	0.89 (0.79–0.95)	169/175	0.97 (0.92–0.99)
TEM	104/106	0.98 (0.93–1)	129/135	0.96 (0.90–0.98)
CTX-M-1-like	70/70	1 (0.94–1)	171/171	1 (0.97–1)
OXA-1	44/44	1 (0.90–1)	197/197	1 (0.98–1)
CMY-2-like	10/10	1 (0.66–1)	228/231	0.99 (0.96–1)
*E. coli* cAmpC	11/155	0.07 (0.04–0.12)	86/86	1 (0.95–1)
KPC	6/6	1 (0.52–1)	233/235	0.99 (0.97–1)
OXA-48	8/8	1 (0.60–1)	232/233	1 (0.97–1)
NDM	15/15	1 (0.75–1)	226/226	1 (0.98–1)
VIM	5/5	1 (0.46–1)	236/236	1 (0.98–1)
ArmA	5/5	1 (0.46–1)	235/236	1 (0.97–1)
RmtB	3/3	1 (0.31–1)	238/238	1 (0.98–1)
RmtC	4/4	1 (0.40–1)	237/237	1 (0.98–1)
RmtF	2/2	1 (0.20–1)	239/239	1 (0.98–1)
AAC(3)-Ia	5/5	1 (0.46–1)	235/236	1 (0.97–1)
AAC(3)-II	40/41	0.98 (0.86–1)	196/200	0.98 (0.95–0.99)
AAC(3)-IV	4/4	1 (0.40–1)	237/237	1 (0.98–1)
AAC(3)-VI	3/3	1 (0.31–1)	238/238	1 (0.98–1)
AAC(6′)-Ib	42/43	0.98 (0.86–1)	197/198	0.99 (0.97–1)
AAC(6′)-Ib-cr	40/40	1 (0.89–1)	200/201	1 (0.97–1)
ANT(2′′)-I	10/11	0.91 (0.57–1)	229/230	1 (0.97–1)
APH(3′)-VI	4/6	0.67 (0.24–0.94)	235/235	1 (0.98–1)
GyrA WT	101/122	0.83 (0.75–0.89)	119/119	1 (0.96–1)
QnrA	4/5	0.80 (0.30–0.99)	236/236	1 (0.98–1)
QnrB	22/26	0.85 (0.64–0.95)	213/215	0.99 (0.96–1)

*CI, confidence interval; WT, wild type.*

For the aminoglycoside resistance mechanisms, i.e., the 16S-RMTases and AMEs, the sensitivity was 100% compared to WGS analysis for each 16S-RTMase, and 100% for the AMEs AAC(3)-Ia, AAC(3)-IV, and AAC(3)-VI. Of the other AMEs, the sensitivity was 97.6% for AAC(3)-II, 97.7% for AAC(6′)-Ib, 90.9% for ANT(2′′)-Ia, and 66.7% for APH(3′)-VI. The specificity was 100% for RmtB, RmtC, RmtF, AAC(3)-IV, AAC(3)-VI, and APH(3′)-VI, 99.6% for ArmA, AAC(3)-Ia and ANT(2′′)-Ia, 98.0% for AAC(3)-II, and 99.5% for AAC(6′)-Ib.

For the fluoroquinolone resistance mechanisms, the sensitivity was 80.0% for QnrA, 84.6% for QnrB, and 100% for AAC(6′)-Ib-cr. The overall sensitivity for the detection of the wildtype QRDR was 82.8% (80.6% in *E. coli* and 86.0% in *K. pneumoniae* and *K. variicola*). The specificity was 100% for the wildtype QRDR, 100% for QnrA, 99.1% for QnrB, and 99.5% for AAC(6′)-Ib-cr. Except for the detection of the peptide defining the wildtype QRDR region of GyrA, no difference in test performance was observed for other resistance mechanisms when comparing the different bacterial species.

### Assay Validation: Discrepancy Analysis

To study the discrepant results between LC-MS/MS and WGS, all false-negative PRM results were reanalyzed manually to distinguish weak or divergent signals from no spectra at all. In the majority of samples with false-negative results, no matching spectra were found. Exceptions were the detected weak or divergent signals for SHV in one isolate, for AAC(6′)-Ib in one isolate, for APH(3′)-VI in one isolate, and for QnrB in two isolates. In all of these isolates, the detected spectra had rdotp values below the cutoff of 0.95 and/or ratios to SIL peptides below the cutoff of 0.01.

Samples with false-positive peptide identifications were both reanalyzed manually and remeasured to differentiate between peptide carryover, detection of background noise or clear spectra, indicating that the corresponding genes may not have been detected by WGS. The false-positive hits for CMY-2-like, KPC, ArmA, AAC(3)-II, and ANT(2′′)-Ia and one false-positive hit for QnrB were shown to be the result of carryover during initial sample analysis, i.e., due to a high-positive sample preceding a negative sample. Furthermore, two identifications of SHV, and one identification of TEM, OXA-48-like, AAC(3)-Ia, and QnrB were not identified during remeasurement and were either due to detection of background noise or caused by sample contamination during initial pretreatment or loading of the Evotips. However, the five other discrepant identifications of TEM, four identifications of SHV, and the identification of AAC(6′)-Ib were all confirmed as remeasurements showed spectra similar to “true positives,” indicating the presence of these resistance mechanisms while the corresponding genes were not detected by WGS.

### Assay Validation: Comparison of Mass Spectrometry Results to Antimicrobial Susceptibility Testing Results

Liquid chromatography-tandem mass spectrometry results were compared with VITEK 2 results to assess how well the developed assay was able to predict (non-)susceptibility to the selected antibiotics (Table 4). Predictions of a susceptible phenotype were in agreement with the VITEK 2 in more than 97.0% of the isolates for imipenem, meropenem, gentamicin, and tobramycin. Furthermore, 93.0% of the predictions of a susceptible phenotype were in agreement with the VITEK 2 for ampicillin, 94.0% for amoxicillin-clavulanic acid, 91.7% for cefotaxime, and 93.7% for ceftazidime. A ciprofloxacin-susceptible phenotype was correctly predicted in 75.7% of the isolates. Predictions of a non-susceptible phenotype were in agreement with VITEK 2 results in 97.4% of the isolates for ampicillin but in only 47.0% of the isolates for amoxicillin-clavulanic acid. Furthermore, predictions were in agreement in 91.0% of the isolates for cefotaxime, 92.3% for ceftazidime, 100% for meropenem, 100% for imipenem, 95.4% for gentamicin, 98.6% for tobramycin, and 93.9% for ciprofloxacin.

**TABLE 4:** Susceptible/non-susceptible predictions for 234 *E. coli*, *K. pneumoniae*, and *K. variicola* isolates based on the detected peptides by LC-MS/MS.

	Isolates phenotypically susceptible				Isolates phenotypically non-susceptible			
		Prediction by LC-MS/MS				Prediction by LC-MS/MS		
Antibiotic	Total (n)	S (n)	NS (n)	Correct (%)	Total (n)	S (n)	NS (n)	Correct (%)
AMP	43	40	3	93.0	191	5	186	97.4
AMC	83	78	5	94.0	151	80	71	47.0
CTX	145	133	12	91.7	89	8	81	91.0
CAZ	143	134	9	93.7	91	7	84	92.3
IPM	205	200	5	97.6	29	0	29	100
MEM	206	200	6	97.1	28	0	28	100
GEN	169	164	5	97.0	65	3	62	95.4
TOB	161	157	4	97.5	73	1	72	98.6
CIP	103	78	25	75.7	131	8	123	93.9

*Results were compared with the categorical interpretations based on VITEK 2 results. Predictions were made for ampicillin (AMP), amoxicillin-clavulanic acid (AMC), cefotaxime (CTX), ceftazidime (CAZ), imipenem (IPM), meropenem (MEM), gentamicin (GEN), tobramycin (TOB), and ciprofloxacin (CIP). S, susceptible; NS, non-susceptible.*

Incorrect non-susceptible predictions (i.e., major errors) for β-lactams and aminoglycosides were partly the result of false positives due to carryover, e.g., carryover of one of the peptides selected for CMY, KPC, or AAC(3)-II. Furthermore, while several identified β-lactamases did result in increased minimum inhibitory concentrations (MICs), the MICs were still within the susceptible category. For instance, *E. coli* cAmpC was identified in nine *E. coli* isolates susceptible to cefotaxime of which three isolates were also susceptible to ceftazidime. In two of these three isolates, MICs were ≤0.12 mg/L and only QPVTQQTLFELGSVSK was detected at a ratio of 0.02 or smaller. In all other cAmpC-positive isolates, this mechanism was detected by both peptides with ratios of at least 0.60 and MICs of ≥1 mg/L were observed for ceftazidime, indicating that cAmpC was expressed but insufficient to result in cefotaxime resistance. Therefore, only the detection of cAmpC peptides without quantification did not allow for accurate prediction of cefotaxime and ceftazidime resistance in these isolates.

Similarly, the presence of OXA-48-like was correctly detected and did result in MICs that were higher than the screening breakpoint for meropenem, but it did not result in resistance to imipenem in three isolates and to meropenem in four isolates, as OXA-48 has limited carbapenemase activity, resulting in a limited increase in MICs for imipenem and meropenem. Amoxicillin-clavulanic acid resistance could not be accurately predicted based on the presence or absence of the present resistance mechanisms. Of the 77 *E. coli* and *K. pneumoniae* complex isolates with SHV and/or TEM as the only identified β-lactamases by LC-MS/MS, 59 isolates were amoxicillin-clavulanic acid resistant. The GyrA QRDR wildtype peptide was false negative in 19 susceptible isolates with QRDR wildtypes, which resulted in non-susceptible predictions. Furthermore, the QRDR wildtype was correctly ruled out in five isolates with either YHPHGD**A**AVYDTIVR, YHPHGD**L**AVYDTIVR, or YHPHGD**L**AVY**N**TIVR, but this did only result in a modest increase in MICs (*n* = 4, MICs 0.12–0.25 mg/L) or no increase at all (*n* = 1, MIC ≤ 0.06 mg/L).

Incorrect susceptible predictions (i.e., very major errors) were mostly caused by resistance mechanisms that were not covered by the included peptides. For instance, *bla*_*CTX*–*M*–14_, *bla*_*CTX*–*M*–27_, and *bla*_*DHA*–1_ also resulted in ampicillin and cephalosporin resistance, and AAC(6′)-Ie-APH(2′′)-Ia was responsible for the only incorrect tobramycin-susceptible prediction. Furthermore, in five ciprofloxacin non-susceptible isolates *qnrS* genes were identified while the resistance mechanisms included in the assay were absent.

### Porin Analysis

Many indicator antibiotics are used to screen for particular mechanisms of resistance. For the absence of porins, no suitable and specific indicator antibiotics are available. Therefore, this mechanism of resistance is often missed. Absence of porins can make organism prone to resistance selection for several antibiotics, including third-generation cephalosporins and carbapenem antibiotics. For this reason, peptides were included to screen for the presence/absence of OmpC and OmpF in *E. coli*. In the majority of *E. coli* isolates, all four peptides of OmpC were detected (*n* = 127). In 21 *E. coli* isolates, OmpC was detected by three peptides and in five isolates by two peptides. In only two isolates, no OmpC peptides were detected, and in one of these isolates, no OmpF peptides were detected either. This specific isolate was amoxicillin-clavulanic acid, cefotaxime, and ceftazidime resistant while no β-lactamases were detected. OmpF was detected by two peptides in 84 of the *E. coli* isolates, by one peptide in 36 of the isolates, and it was not detected by either peptide in 35 isolates.

## Discussion

Currently used growth inhibition techniques delay the time to final AST results of positive blood cultures (Florio et al., 2018; Lamy et al., 2020). In turn, this can delay the time to optimal therapy in septic patients (Timbrook et al., 2017; Babowicz et al., 2021). The use of rapid diagnostic tests has been associated with a reduction in mortality (Timbrook et al., 2017; Guillamet et al., 2019). In addition, rapid diagnostic tests can result in more timely infection prevention measures, in shorter hospital stays, and it can save costs (Kerremans et al., 2008; Pliakos et al., 2018). Commercially available molecular tests such as BioFire FilmArray BCID2 (BioFire) and Verigene BC-GN (Luminex) offer a shorter TAT but only cover a limited number of targets in Gram-negative organisms, and several β-lactamases, and various aminoglycoside and fluoroquinolone resistance mechanisms are not included. WGS is sometimes cited as a future diagnostic tool for molecular resistance testing that is not limited by the number of targets; however, the TAT of WGS is too long and analysis is still costly (Ellington et al., 2017). Furthermore, both WGS and previously mentioned nucleic acid tests are unable to detect the protein presence or protein quantity of resistance mechanisms. Lateral flow assays can be used for the rapid detection of several β-lactamase proteins in blood cultures, but currently available assays only cover a few enzymes (Bodendoerfer et al., 2019). Targeted LC-MS/MS is an alternative technique that can also be applied for the accurate detection and quantification of resistance mechanisms (Charretier and Schrenzel, 2016). In addition, LC-MS/MS can also be used for the identification of bacteria by identifying species-specific peptides using a targeted or untargeted approach (Charretier et al., 2015a; Roux-Dalvai et al., 2019). In theory, a future combination of both applications could result in an all-in-one diagnostic tool for the identification of bacteria and determination of resistance mechanisms. While the ability of targeted LC-MS/MS to accurately and rapidly identify several resistance mechanisms in bacterial subcultures has been demonstrated in previous studies (Charretier et al., 2015a,b; Hassing et al., 2016; Wang et al., 2017, 2019a,b; Cecchini et al., 2018; Foudraine et al., 2019; Strich et al., 2019), to our knowledge, no studies have demonstrated its use for the direct detection of resistance mechanisms in positive blood cultures. Furthermore, most studies focused on a limited number of AMR mechanisms, and not on the prediction of AMR in prospectively collected cultures.

In this study, the use of targeted LC-MS/MS for the direct detection of various classes of resistance mechanisms in positive blood cultures was demonstrated, with an emphasis on mechanisms frequently present in *E. coli* and *K. pneumoniae*. Specific marker peptides were selected using an *in silico* genoproteomic approach followed by a simple evaluation using MS or they were selected from previous studies (Foudraine et al., 2019, 2021a,^2022^). All selected peptides were added to a single multiplex panel for the detection of 10 β-lactamases, 8 AMEs, 4 16S-RMTases, 2 Qnr proteins, the wildtype QRDR of GyrA, the 2 main porins in *E. coli*, and 3 internal quality control proteins to confirm proper sample pretreatment. During assay development, several assay characteristics were prioritized. Desired key characteristics were a short time to result, a simple sample pretreatment protocol, a short analysis time per sample, a substantial number of targets, a high accuracy in detecting the presence or absence of the selected AMR mechanisms, and automated result analysis.

Overall, the correct phenotype, i.e., susceptible or non-susceptible, was correctly predicted based on LC-MS/MS results in more than 95% of the isolates for ampicillin, imipenem, meropenem, gentamicin, and tobramycin, while the predictions were correct in more than 90% of the isolates for cefotaxime and ceftazidime. Predictions were overall less accurate and unsatisfactory for ciprofloxacin (85.9% of isolates correctly classified) and amoxicillin-clavulanic acid (67.5% of the isolates correctly classified). For ciprofloxacin, mainly the lacking sensitivity for the QRDR wildtype hampered the predictive value. In contrast, amoxicillin-clavulanic acid resistance could not be predicted accurately based on only the presence or absence of the selected resistance mechanisms. Most likely TEM and SHV hyperproduction, and inhibitor-resistant TEM and SHV were responsible for amoxicillin-clavulanic acid resistance in the isolates with incorrect susceptible predictions (Perez-Moreno et al., 2004).

In this assay, a high multiplexing capacity and a short gradient were prioritized over quantitative performance and peptides with a high sensitivity were selected that are not necessarily the most suitable peptides for quantification. Nevertheless, quantification capabilities of the assay should be further evaluated as quantification is important not only to distinguish TEM and SHV hyperproducers but also to better predict (non)-susceptibility based on the quantity of porins and efflux pumps. Other ways to improve the assay include a simple column rinse step between each sample to diminish possible carryover from samples containing high concentrations of a selected peptide. Furthermore, the time to result and hands-on time could be further reduced through automation of sample pretreatment. In addition, peptides for CTX-M-9-like β-lactamases (including CTX-M-14 and CTX-M-27) should be added to the current panel. The predictive value for ciprofloxacin (non-)susceptibility should also be increased. Perhaps, longer digestion times or an increase in detection time would increase the sensitivity for detection of the wildtype QRDR of GyrA. In a previous study of Hassing et al. (2016) it was already shown that a 100% sensitivity and specificity for the detection of wildtype and mutated QRDRs can be achieved in *Salmonella enterica* isolates using LC-MS/MS. In that study, a more elaborate sample pretreatment procedure was used, including acetone precipitation, reduction, and alkylation steps and an overnight digestion step (Hassing et al., 2016). Further, a 30 min gradient was used for the detection of 12 peptides, while in this study, 56 endogenous and 56 SIL peptides were measured using a gradient of only 11.5 min.

During assay development, candidate peptides were also selected *in silico* for QnrS and for the wildtype QRDR of ParC. However, both mechanisms could not be detected using the current protocol. Unfortunately, the QRDR of ParC is a relatively long peptide consisting of 23 amino acids and contains a cysteine amino acid. Because of the latter reason, detection requires reduction and alkylation steps during pretreatment, steps that we omitted from the current protocol to shorten the time to result.

While we were unable to detect these mechanisms due to the aforementioned reasons, several other resistance mechanisms were detected by LC-MS/MS that were missed using growth inhibition techniques. For example, the production of cAmpC was detected in one isolate with a ceftazidime MIC that was still within the susceptible category. Other examples were the detection of OXA-48 in three imipenem susceptible isolates and absence of the wildtype QRDR that correctly indicated mutations in five isolates that were still susceptible to ciprofloxacin but that are prone for resistance selection once exposed to a quinolone (Robicsek et al., 2006). These discrepancies highlight the fact that LC-MS/MS can be used as both a screening and confirmatory tool for the detection of resistance mechanisms that are easily missed using growth inhibition techniques. Ideally, presence of these resistance mechanisms should be taken into account when selecting the most optimal treatment (Robicsek et al., 2006; Rodriguez-Bano et al., 2018; Stewart et al., 2018). As opposed to WGS, the developed LC-MS/MS assay could also discern the *E. coli* isolates that produce chromosomal encoded AmpC (*n* = 11) from all other *E. coli* isolates that only carry the gene (*n* = 144); however, this was not confirmed using a different protein detection technique. Limitations of this study were the limited number of prospectively collected blood cultures in general and the limited number of polymicrobial cultures in particular. Furthermore, the assay was only developed and validated for the detection of key resistance mechanisms present in *E. coli* and *K. pneumoniae*. For the detection of the most prevalent resistance mechanisms in other bacterial species such as *S. aureus*, *A. baumannii*, and *P. aeruginosa*, different peptides would be required. Finally, discrepancies between LC-MS/MS and VITEK2 results, or between LC-MS/MS and WGS results, could have been studied in more detail using, for instance, additional broth microdilution susceptibility tests and/or targeted PCRs. General limitations of the technique were still the limited number of targets that can be detected when using a targeted approach with a gradient of only 11.5 min and the significant (one-time) investment when purchasing an Orbitrap mass spectrometer.

In conclusion, a targeted LC-MS/MS workflow was developed that could detect various β-lactam, aminoglycosides, and fluoroquinolone resistance mechanisms. (Non-)susceptibility predictions were accurate for most of the tested antibiotics and the assay had a time to result of approximately 3 h. This study shows that LC-MS/MS can be applied for rapid detection of bacterial resistance mechanisms in positive blood cultures. Future work should focus on assay automation, laboratory implementation, and demonstration of patient benefit and cost-effectiveness. Finally, extra peptides can be included for resistance mechanisms against additional antimicrobials such as trimethoprim-sulfamethoxazole, and for resistance mechanisms of other bacterial species to make the developed workflow more universally applicable.

## Data Availability Statement

The datasets presented in this study can be found in online repositories. The names of the repository/repositories and accession number(s) can be found in the article/supplementary material. A table with all isolates and their genomic and proteomic identifiers is available as supplemental dataset S1. The genomic sequencing data of the isolates collected from blood cultures is available in the ENA repository under the primary accession number PRJEB44242 and secondary accession number ERP128275. The mass spectrometry proteomics data have been deposited to the ProteomeXchange Consortium via the PRIDE (Perez-Riverol et al., 2019) partner repository with the dataset identifier PXD025365. The “Antibiotic Resistance Scanner” software is freely available upon request (https://www.davinci-ls.com/en/contact/contact-head-offices).

## Author Contributions

WG, TL, DF, and LD designed the study. SN, MR, WK, DF, and LD performed the laboratory work. DF, SN, NS, and LD analyzed the data. PC developed software that was used in this study. NS and LD curated the data in online repositories. WG, TL, CK, and AV supervised the study. WG and TL obtained project funding. WG performed the project administration. DF wrote the first version of the manuscript. All authors edited and approved the manuscript.

## Conflict of Interest

The authors declare that the research was conducted in the absence of any commercial or financial relationships that could be construed as a potential conflict of interest.

## Publisher’s Note

All claims expressed in this article are solely those of the authors and do not necessarily represent those of their affiliated organizations, or those of the publisher, the editors and the reviewers. Any product that may be evaluated in this article, or claim that may be made by its manufacturer, is not guaranteed or endorsed by the publisher.

## Footnotes

1
https://github.com/tseemann/abricate

2
https://www.eucast.org/expert_rules_and_intrinsic_resistance/

3
http://vassarstats.net
